# Dissatisfaction of people with type 2 diabetes with the care received at a diabetes clinic in Ningbo, China: A cross‐sectional study

**DOI:** 10.1002/hsr2.927

**Published:** 2022-10-31

**Authors:** Jingjia Yu, Jialin Li, Miao Xu, Li Li, Kaushik Chattopadhyay

**Affiliations:** ^1^ Department of Endocrinology and Metabolism Ningbo First Hospital Ningbo People's Republic of China; ^2^ Lifespan and Population Health Academic Unit, School of Medicine University of Nottingham Nottingham UK

**Keywords:** China, dissatisfaction, medical care, type 2 diabetes mellitus

## Abstract

**Aim:**

The study aimed to assess the dissatisfaction of people with type 2 diabetes mellitus (T2DM) with the care that they received at a diabetes outpatient clinic in Ningbo, China and to determine the associated factors.

**Methods:**

A cross‐sectional study was conducted among 406 adults with T2DM in 2020–2021. Those who were treated at the diabetes outpatient clinic for at least six consecutive months before the survey date were eligible. The Short Assessment of Patient Satisfaction scale was used to assess participants' dissatisfaction with the care that they received.

**Results:**

Of the participants, 25.1% were not satisfied with the care that they received at the diabetes outpatient clinic in Ningbo. The odds of dissatisfaction were higher in physically active people compared to those who were not (odds ratio [OR]: 3.41; 95% confidence interval [CI]: 1.56–7.45) and those with >1–5 years of T2DM compared to ≤1 year (OR: 2.18; 95% CI: 1.05–4.53).

**Conclusion:**

A quarter of people with T2DM were dissatisfied with the care that they received at the diabetes outpatient clinic in Ningbo, China, and the factors associated with dissatisfaction were identified.

## 1 INTRODUCTION

Diabetes mellitus is one of the most common chronic diseases, and its global prevalence is high and increasing.^1^ The majority of adults currently diagnosed have type 2 diabetes mellitus (T2DM), and a huge population with T2DM is currently undiagnosed.^1^ T2DM is a complex metabolic disorder, and chronic hyperglycemia in T2DM is associated with macrovascular complications (e.g., coronary heart disease, stroke, and peripheral arterial disease), microvascular complications (e.g., diabetic retinopathy, nephropathy, and neuropathy/foot), and even death.^1^, ^2^ Apart from these significant health consequences, the socioeconomic impact of the disease is also grave.^1^, ^2^

The prevalence of diabetes is high in Chinese adults.^3^ In other words, more than 140 million adults in China are living with diabetes.^4^ The increasing prevalence of T2DM and its health, social, and economic consequences are major concerns in China.^5^, ^6^ Ningbo, located in the northeast of China, is one of the most economically developed cities, and around 21% of people over 40 years of age have T2DM.^7^ In China, there is no referral system and people are allowed to attend any hospital and clinic of their choice.^8^

No permanent cure exists for T2DM, and high‐quality care should be provided to people with T2DM throughout their lifespan.^9^ This high‐quality care should be comprehensive and include routine check‐ups to enforce lifestyle modification, prescribe and adjust medication, and detect and manage any T2DM‐related complications and comorbidities.^10^ Patients' views, such as satisfaction with the treatment provided, explanation of treatment results, clinician care, participation in medical decision‐making, respect by the clinician, time with the clinician, and satisfaction with the hospital or clinic care, are important.^11^ The dissatisfaction of people with T2DM with the care that they received can have a major negative impact, such as poor self‐efficacy and nonadherence to therapy and ultimately leading to poorer outcomes like poor glycemic control and T2DM complications.^12^ Thus, there is a need to explore dissatisfaction in patients and address their concerns as part of health service improvement.^11^ However, limited research has been conducted on this issue in other parts of China.^13^, ^14^ Therefore, the study aimed to assess the dissatisfaction of people with T2DM with the care that they received at a diabetes outpatient clinic in Ningbo, China and to determine the associated factors.

## 2 METHODS

### 2.1 Study design, site, and period

A cross‐sectional study was conducted at a diabetes outpatient clinic in the Department of Endocrinology and Metabolism, Ningbo First Hospital, China from November 1, 2020 to May 31, 2021.

### 2.2 Study participants and eligibility criteria

The study included people with T2DM, aged 18 years or above. The T2DM diagnosis was based on the Chinese guideline.^15^ People who were treated at the diabetes outpatient clinic for at least six consecutive months before the survey date and gave written informed consent to participate in the study were included. T2DM being a chronic disease, six months' time is useful to adjust to the treatment and reflect on the care received.^8^

### 2.3 Data collection tool and procedure

A quantitative questionnaire in Mandarin was used for data collection. The self‐reported nonstandardized questions were developed and pretested in six people with T2DM (not included in this study). Five trained nurses collected and entered the data. A doctor was responsible for checking the data quality. Data on the following variables were collected (and categorized): age (18–39, 40–59, or ≥60 years), sex (male or female), education (university/college, class 7–12, class 1–6, or no qualification), occupation (manual worker, nonmanual worker, or retired/never worked), marital status (married or single/divorced/widowed), residence (urban or rural; based on the “hukou” residence registration system in China^16^), health insurance (yes or no), physically active (yes or no; current status of doing moderate or high physical activity, assessed using the Mandarin version of the International Physical Activity Questionnaire—short format^17^), healthy diet (yes or no; current status, more than 50% A + B answers [i.e., healthy dietary choices] on the adapted version of the UK Diabetes and Diet Questionnaire^18^), smoker (no or yes; current status), alcohol drinker (no or yes; current status), family history of T2DM (no or yes; T2DM in any parent or sibling), duration of T2DM (≤1, >1–5, >5–10, or >10 years), glycated hemoglobin (HbA1c) in control (yes or no; HbA1c < 7% was considered as in control,^15^, ^19^ assessed using venous blood sampling and the high‐performance liquid chromatographic method; D‐10 Hemoglobin Analyzer, Bio‐Rad), overweight/obesity (no or yes; body mass index ≥ 24.0 kg/m^2^ was considered as overweight/obesity,^20^ calculated as body weight in kg divided by height in m^2^, body weight [to the nearest 0.1 kg] and height [to the nearest 0.5 cm] were measured with light clothes and without shoes in standing position using a calibrated automatic digital weight and height scale; HNH‐318, Omron, Japan), and hypertension (no or yes; blood pressure ≥ 140/90 mmHg was considered as hypertension,^21^ measured using a calibrated, automated sphygmomanometer). The anthropometric and physiological parameters were measured twice per participant, and the average of these two readings was used in the analysis. The Short Assessment of Patient Satisfaction scale was used to assess participants' dissatisfaction with the care that they received.^11^ It is a seven‐item scale that focuses on treatment satisfaction, explanation of treatment results, clinician care, participation in medical decision‐making, respect by the clinician, time with the clinician, and satisfaction with clinic care. The total score starts from 0 (very dissatisfied) to 28 (very satisfied). For this study, very dissatisfied (0–10) and dissatisfied (11–18) were combined as dissatisfied. Similarly, very satisfied (27–28) and satisfied (19–26) were combined as satisfied.

### 2.4 Sample size

At least 377 participants were required in the study based on a 95% confidence level (CI) and a 5% margin of error.^22^ Consecutive people with T2DM were approached and recruited 406 participants.

### 2.5 Ethics approval

Ethics approval was received from the Research Ethics Committee of Ningbo First Hospital. The participant information sheet and consent form were available in Mandarin. The study objective was explained to all the eligible participants, and written informed consent was taken from those interested in participating.

### 2.6 Statistical analyses

We calculated the number and percentage for categorical variables and mean and standard deviation (SD) for normally distributed continuous data. A *χ* ^2^ test was conducted, and the result was considered statistically significant when the *p* value was ≤0.05. We developed a multiple logistic regression model to determine the factors independently associated with dissatisfaction, and for this, we used the backward stepwise regression analysis and included all the variables. A sensitivity analysis was carried out, and only those variables with a *p* value of ≤0.20 obtained through the *χ* ^2^ test were included in the multiple logistic regression model. The models included a sample with unknown values for these variables. The odds ratio (OR) and 95% CI were calculated. We used IBM SPSS Statistics version 23.0 for Windows for data analyses.

## 3 RESULTS

Participant characteristics are reported in Table 1. The mean age of participants was 56.3 years (SD ± 12.6), and 62.6% were men. Of the participants, 25.1% were not satisfied with the care that they received at the diabetes outpatient clinic in Ningbo, China, and physically active (*p* = 0.003) and duration of T2DM (*p* = 0.037) were found to be associated with it.

**Table 1:** Characteristics of study participants

	Satisfied	Dissatisfied	*p* value
	*n* (%)	*n* (%)	
	304 (74.9)	102 (25.1)	
Age (years)			0.912
18–39	39 (12.8)	13 (12.7)	
40–59	133 (43.8)	47 (46.1)	
≥60	132 (43.4)	42 (41.2)	
Sex			0.451
Male	187 (61.5)	67 (65.7)	
Female	117 (38.5)	35 (34.3)	
Education			0.881
University/college	42 (13.8)	15 (14.7)	
Class 7–12	162 (53.3)	51 (50.0)	
Class 1–6	70 (23.0)	27 (26.5)	
No qualification	30 (9.9)	9 (8.8)	
Occupation			0.586
Manual worker	57 (18.7)	21 (20.6)	
Nonmanual worker	92 (30.3)	35 (34.3)	
Retired/never worked	155 (51.0)	46 (45.1)	
Marital status			0.811
Married	288 (94.7)	96 (94.1)	
Single/divorced/widowed	16 (5.3)	6 (5.9)	
Residence			0.180
Urban	183 (60.2）	69 (67.6)	
Rural	121 (39.8）	33 (32.4)	
Health insurance			0.877
Yes	291 (95.7)	98 (96.1)	
No	13 (4.3)	4 (3.9)	
Physically active			0.003
Yes	240 (78.9)	94 (92.2)	
No	64 (21.1)	8 (7.8)	
Healthy diet			0.296
Yes	291 (95.7)	95 (93.1)	
No	13 (4.3)	7 (6.9)	
Smoker			0.473
No	237 (78.0)	76 (74.5)	
Yes	67 (22.0)	26 (25.5)	
Alcohol drinker			0.929
No	216 (71.1)	72 (70.6)	
Yes	88 (28.9)	30 (29.4)	
Family history of T2DM			0.136
No	165 (54.3)	64 (62.7)	
Yes	139 (45.7)	38 (37.3)	
Duration of T2DM (years)			0.037
≤1	55 (18.1)	15 (14.7)	
>1–5	56 (18.4)	32 (31.3)	
>5–10	63 (20.7)	22 (21.6)	
>10	130 (42.8)	33 (32.4)	
HbA1c in control			0.759
Yes	83 (27.3)	31 (30.4)	
No	216 (71.1)	70 (68.6)	
Unknown	5 (1.6)	1 (1.0)	
Overweight/obesity			0.224
No	147 (48.4)	49 (48.0)	
Yes	157 (51.6)	52 (51.0)	
Unknown	0 (0)	1 (1.0)	
Hypertension			0.820
No	220 (72.4)	75 (73.5)	
Yes	84 (27.6)	27 (26.5)	

Abbreviations: HbA1c, glycated hemoglobin; T2DM, type 2 diabetes mellitus.

Table 2 shows the multiple logistic regression analysis to determine the factors independently associated with dissatisfaction. The odds of dissatisfaction were higher in physically active people compared to those who were not (OR: 3.41; 95% CI: 1.56–7.45) and those with >1–5 years of T2DM compared to ≤1 year (OR: 2.18; 95% CI: 1.05–4.53). Similar results were found in the sensitivity analysis.

**Table 2:** Multiple logistic regression analysis to determine the factors independently associated with dissatisfaction

	OR (95% CI)
Physically active	
No	1
Yes	3.41 (1.56–7.45)
Duration of T2DM (years)	
≤1	1
>1–5	2.18 (1.05–4.53)
>5–10	1.26 (0.59–2.69)
>10	0.88 (0.44–1.77)

Abbreviations: CI, confidence interval; OR, odds ratio; T2DM, type 2 diabetes mellitus.

## 4 DISCUSSION

Around 25% of participants in this study were not satisfied with the care that they received at the diabetes outpatient clinic in Ningbo, China. This is consistent with the worldwide literature on patient satisfaction, including people with T2DM, which shows that around 10%–30% of patients are dissatisfied with their health care.^11^, ^23^, ^24^, ^25^ Studies conducted in other parts of China among people with T2DM found moderate levels of satisfaction (mean scores: 3.14 out of 5 and 34.54 out of 60).^13^, ^14^ A direct comparison between the findings should be avoided as different questionnaires were used in the studies—one had a short question on satisfaction and the other used a different standardized questionnaire.^13^, ^14^ A qualitative study conducted among people with T2DM and general practitioners in Ningbo found several issues related to the diagnosis and management of T2DM in primary care, including unacceptable variations in care.^26^

In our study, the odds of dissatisfaction were higher in physically active people compared to those who were not physically active. The finding is inconsistent with other studies conducted among people with T2DM in China, and they found that those who were doing regular exercise were more satisfied.^13^, ^14^ This could be due to the fact that physically active people in our study (around 82%) thought that they already knew how to lead a healthy lifestyle and there was nothing new or more for them in the care that they received at the diabetes clinic. In this study, around 70% of people had poor glycemic control, but this was not related to low physical activity (additional data analysis). This could be another reason for dissatisfaction as physically active people were unable to see the benefits. However, dissatisfaction was not associated with HbA1c levels in this study and other studies conducted in China and elsewhere.^13^, ^24^ A qualitative study should be conducted among them to explore the reasons behind such dissatisfaction.

In our study, the odds of dissatisfaction were higher in people with >1–5 years of T2DM compared to ≤1 year. A study conducted among people with T2DM with inadequate glycemic control in Romania found that those with >5 years of T2DM had higher satisfaction levels compared to those with 5–10 years and <10 years of T2DM.^27^ This could be due to the different reference groups or populations, settings, and contexts. People with newly diagnosed T2DM in Ningbo might have lower expectations, and they appreciated whatever care they received at the diabetes clinic. However, those who had T2DM for several years were not satisfied, which could be due to multiple reasons, including boredom with the care that they received or not receiving high‐quality care in subsequent years. The longer duration of T2DM is found to be associated with poor glycemic control, high T2DM‐related complications and comorbidities (and polypharmacy), and low health‐related quality of life;^8^, ^28^, ^29^, ^30^ all these issues advocate the need for long‐term high‐quality care. Again, a qualitative study should be able to shed light on the reasons behind such dissatisfaction.

The study has several strengths and weaknesses. To the best of the authors' knowledge, this was the first study on this issue in Ningbo and more widely in the Zhejiang Province in which Ningbo city is located. In terms of generalizability, the findings could be valid in similar populations, settings, and contexts. Missing data on adjusted variables were extremely low in the study, and the multiple logistic regression analysis included the sample with missing data. Data on many variables were self‐reported, and recall bias and social desirability bias could have been an issue, for example, duration of T2DM and healthy lifestyle, respectively. A better approach would be using medical records and objective measures. Since the study was cross‐sectional and not longitudinal, the causal relationship between the adjusted variables and dissatisfaction could not be determined. The associations found in the study could be due to other factors not adjusted for in the multiple logistic regression model, such as waiting time outside the clinic, T2DM therapeutic regimen (e.g., oral antidiabetic drugs, insulin), and other T2DM‐related complications and comorbidities.^24^, ^31^, ^32^

In conclusion, a quarter of people with T2DM were dissatisfied with the care that they received at the diabetes outpatient clinic in Ningbo, China, and the factors associated with dissatisfaction were identified. The findings support the need to address patients' concerns as part of health service improvement.

## AUTHOR CONTRIBUTIONS

**Jingjia Yu**: Formal analysis; writing – original draft; writing – review and editing. **Jialin Li**: Conceptualization; funding acquisition; investigation; methodology; project administration; supervision; writing – review and editing. **Miao Xu**: Project administration; supervision; writing – review and editing. **Li Li**: Conceptualization; funding acquisition; investigation; project administration; supervision; writing – review and editing. **Kaushik Chattopadhyay**: Conceptualization; formal analysis; funding acquisition; investigation; methodology; project administration; supervision; writing – original draft; writing – review and editing.

## CONFLICT OF INTEREST

The authors declare no conflict of interest.

## TRANSPARENCY STATEMENT

The lead author Kaushik Chattopadhyay affirms that this manuscript is an honest, accurate, and transparent account of the study being reported; that no important aspects of the study have been omitted; and that any discrepancies from the study as planned (and, if relevant, registered) have been explained.

## Data Availability

The data set will be available upon request unless there are legal or ethical reasons for not doing so.
